# Characterization of a new oxidant-stable serine protease isolated by functional metagenomics

**DOI:** 10.1186/2193-1801-2-410

**Published:** 2013-08-28

**Authors:** Sophie Biver, Daniel Portetelle, Micheline Vandenbol

**Affiliations:** Unité de Microbiologie et Génomique, Gembloux Agro-Bio Tech, Université de Liège, Avenue Maréchal Juin 6, B-5030 Gembloux, Belgium

**Keywords:** Calcium, Functional metagenomics, Oxidant, Serine protease

## Abstract

A novel serine protease gene, *SBcas3.3*, was identified by functional screening of a forest-soil metagenomic library on agar plates supplemented with AZCL-casein. Overproduction in *Escherichia coli* revealed that the enzyme is produced as a 770-amino-acid precursor which is processed to a mature protease of ~55 kDa. The latter was purified by affinity chromatography for characterization with the azocasein substrate. The enzyme proved to be an alkaline protease showing maximal activity between pH 9 and 10 and at 50°C. Treatment with the chelating agent ethylenediaminetetraacetic acid irreversibly denatured the protease, whose stability was found to depend strictly on calcium ions. The enzyme appeared relatively resistant to denaturing and reducing agents, and its activity was enhanced in the presence of 10 ml/l nonionic detergent (Tween 20, Tween 80, or Triton X-100). Moreover, SBcas3.3 displayed oxidant stability, a feature particularly sought in the detergent and bleaching industries. SBcas3.3 was activated by hydrogen peroxide at concentrations up to 10 g/l and it still retained 30% of activity in 50 g/l H_2_O_2_.

**Electronic supplementary material:**

The online version of this article (doi:10.1186/2193-1801-2-410) contains supplementary material, which is available to authorized users.

## Introduction

Microorganisms produce various enzymes and antimicrobial agents potentially useful in agriculture, the chemical industry, and both human and veterinary medicine (Daniel 2004). Among these are proteases, ubiquitous enzymes playing vital roles in most cellular processes, being required mainly for protein turnover and maturation and also for nutrition (Rao et al. 1998). As their ability to hydrolyse the peptide bonds of various proteinaceous substrates has attracted the interest of industrialists in several fields, proteases are among the most important enzymes in industrial use. The alkaline proteases used in the detergent industry represent the largest share of the protease market. They are present mainly in laundry detergents, where they help remove protein stains such as blood, grass, or milk from clothing. They also find various applications in the leather, photographic, and pharmaceutical industries and in the management of industrial and household wastes. Furthermore, they are widely used in the food industry, notably for cheese making, baking, preparation of soy hydrolysates, and meat tenderizing (Fujinami and Fujisawa 2010; Gupta et al. 2002; Rao et al. 1998; Saeki et al. 2007).

Until a few years ago, industrially interesting microbial enzymes could be isolated only from microorganisms cultivable by standard laboratory techniques. It has been estimated, however, that these organisms represent less than 1% of the total number of microbial species in soil samples (Torsvik et al. 1998; Torsvik et al. 1990). Today, with the development of functional metagenomics, it is possible to tap additionally the vast and heretofore unexploited reservoir of natural products produced by 'uncultivable' microorganisms, to discover new enzymes with better-suited properties or for new biotechnological applications. The approach consists in cloning total DNA from an environmental sample in an easily cultivable host, usually *Escherichia coli*, and screening the metagenomic libraries obtained for a desired activity on medium supplemented with a substrate of the type of enzyme sought (Daniel 2005). This strategy has been applied to several ecosystems, allowing detection of diverse enzymes such as cellulases, xylanases, glucosidases, amylases, and esterases (Duan and Feng 2010; Kennedy et al. 2011; Nimchua et al. 2012;Rondon et al. 2000).

To date, only a few proteases have been isolated by functional metagenomics (Lee et al. 2007; Neveu et al. 2011; Pushpam et al. 2011). Here, a forest-soil library previously screened with success for lipolytic and antimicrobial activities was used to search for new proteases. This has led to isolation of a new alkaline serine protease, which we have purified and biochemically characterized.

## Results

### Identification of a new protease gene by functional screening

Two forest-soil metagenomic libraries were screened in *Escherichia coli* to find new interesting proteolytic activities. Both plasmid libraries, differing only in their average insert size (7 and 12 kb), had been successfully screened for lipolytic (Biver and Vandenbol 2013) and antimicrobial activities (Biver et al. 2013). Further analysis of the small-insert library allowed us to identify one positive clone on agar plates supplemented with 1 g/l AZCL-casein. Casein hydrolysis was not observed after the 2-day incubation at 37°C, but appeared after about a week of additional incubation at room temperature (~22°C). Sequence analysis of the 3,889-bp insert (62% GC) responsible for the proteolytic activity revealed an open reading frame of 2,310 bp (770 amino acids) showing homology to various serine proteases. The new enzyme, named SBcas3.3, was most similar (69% similarity, 54% amino acid identity over 770 aa) to an uncharacterized protease of *Desulfobacter postgatei* 2ac9 (ZP_10168560.1).

**Figure 1:**
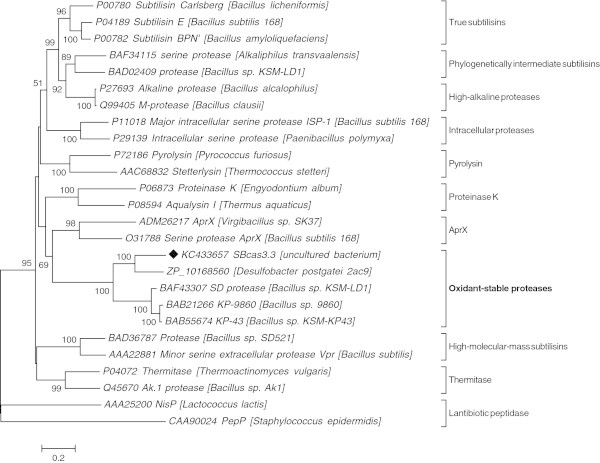
**Neighbour-joining tree built with SBcas3.3 and members of the S8A subfamily of serine proteases.** Members of the S8 family of serine proteases (also known as the subtilase family) have been classified into six groups: subtilisin, thermitase, proteinase K, lantibiotic peptidase, pyrolysin (grouped into the S8A subfamily in the MEROPS database) and kexin (S8B subfamily, not shown on this figure) (Siezen and Leunissen 1997). Subtilisins have been further classified into six subfamilies, namely true subtilisins, high-alkaline proteases, intracellular proteases, phylogenetically intermediate subtilisins between true subtilisins and high-alkaline proteases, high-molecular-mass subtilisins, and oxidant-stable proteases. The phylogenetic analysis shown here was done with SBcas3.3, the protease to which SBcas3.3 is the most similar (ZP_10168560: regulatory P domain of subtilisin-like proprotein convertases, *Desulfobacter postgatei*) and representative members of each of the S8A groups mentioned above, as well as members of the AprX subfamily (Phrommao et al. 2011) (a new group whose position inside the S8A protease family is not well defined). The Poisson correction method was used to compute evolutionary distances. The scale bar indicates the number of amino acid substitutions per site. Bootstrap values are expressed as percentages of 1,000 replications and are shown at the nodes. Only bootstrap values higher than 50% are indicated.

**Figure 2:**
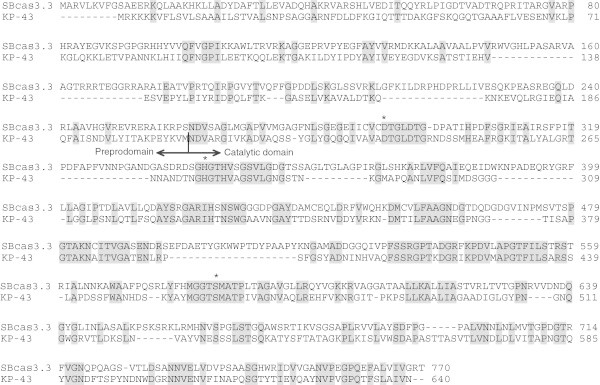
**Sequence alignment between SBcas3.3 and the KP-43 peptidase of*Bacillus*sp. strain KSM-KP43 (BAB55674).** Conserved residues are shaded in grey. Asterisks: conserved catalytic residues. The vertical bar indicates separation between the preprodomain of KP-43 and its catalytic domain released after autocatalytic cleavage.

A BlastP search against the MEROPS peptidase database (Rawlings et al. 2012) revealed that SBcas3.3 belongs to the S8A subfamily of serine proteases (Figure 1), a vast group of enzymes related to subtilisin Carlsberg, an endopeptidase of *Bacillus licheniformis*. These enzymes are characterized by the active site residues (D, H, S; D291, H342, S584 for SBcas3.3) located in the motifs **D**-(T/S)-G, **H**-G-T-H, and G-T-**S**-M-A-X-P. Within this subfamily, the biochemically characterized protease to which SBcas3.3 is most similar (49% similarity, 33% identity over 688 aa) is the alkaline protease KP-43 isolated from *Bacillus* sp. strain KSM-KP43 (Saeki et al. 2002) (Figure 2). Unlike most subtilisin-like proteases, KP-43 and its few homologues (MEROPS ID: S08.123) have been shown to be highly resistant to oxidants, which makes them attractive for use in bleach-based detergents (Saeki et al. 2007).

### Protein overproduction in *E. coli* Rosetta cells

**Figure 3:**
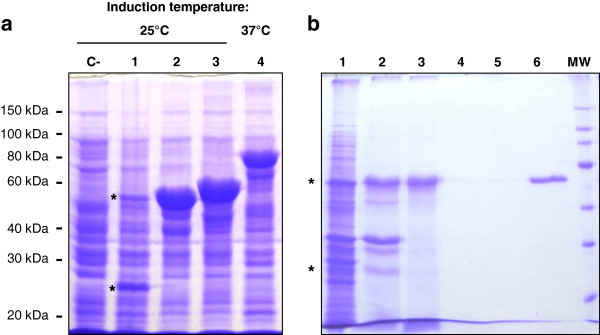
**SDS-PAGE analysis of the recombinant protease. a**. Total extracts of *E. coli* Rosetta cells expressing SBcas3.3 or one of its two artificially N-truncated variants (cas516 or cas560). C-: Total extract of induced *E. coli* Rosetta 2 (DE3) cells transformed with the empty vector pET-30b(+); lanes 1 and 4: total extracts of cells overproducing SBcas3.3 at 25°C (lane 1, cleaved protease, the asterisks indicate the two parts of the protein) or 37°C (lane 4, immature full-length protease); lanes 2 and 3: total extracts of cells overproducing N-truncated forms of the protease (cas516 and cas560). **b**. Purification of the mature protease. Proteins recovered during different purification steps were separated by SDS–10% polyacrylamide gel electrophoresis and stained with Coomassie Brilliant Blue. Lane 1: total cell extract of induced bacteria overproducing the mature form of SBcas3.3 (the asterisks indicate the two parts of the protein); lane 2: insoluble fraction of the cell extract containing the mature protease; lane 3: soluble fraction obtained after treatment of the insoluble fraction with urea and Triton X-100; lanes 4 and 5: flow-throughs 1 and 2; lane 6: purified protein; MW: protein molecular weight markers.

In order to purify the protease, its coding sequence was cloned in fusion with a sequence encoding a C-terminal His-tag in the IPTG-inducible pET-30b(+) vector allowing overexpression in *E. coli*. After induction for 16 h at 37°C, the bacteria were harvested and lysed to extract soluble and insoluble proteins, which were then analysed by SDS-PAGE, together with the cell culture supernatants (data not shown). The proteolytic activity of each collected fraction was evaluated at several pH values (4–10) and temperatures (4-50°C) (data not shown). SBcas3.3 was found exclusively in the insoluble fraction, as an inactive protein migrating as a single band corresponding to a molecular mass of ~80 kDa, close to its theoretical molecular mass of 83 kDa (Figure 3a). When cells were grown at lower temperatures (30, 25, or 16°C) in an attempt to reduce inclusion body formation, the 80-kDa band disappeared in favour of two smaller fragments of approximately 27 and 55 kDa (Figure 3a). Although the protein was cleaved and also found exclusively in the insoluble fraction, it was active since proteolytic activity was observed between pH 6 and 10 (optimal pH ~9) at temperatures below 37°C (maximal activity around 25°C). As western blot analysis with anti-His-tag antibodies revealed that only the 55-kDa fragment contains the C-terminal His-tag (data not shown), it was tempting to speculate that the 27-kDa fragment might be the prodomain of SBcas3.3, the two fragments of the protein resulting probably from an autocatalytic cleavage required for protease maturation. Many proteases are synthesized as larger, inactive precursors or (pre)proproteins, whose prodomains function as dedicated intramolecular chaperones and sometimes also as potent protease inhibitors regulating precise activation of their cognate catalytic domains (Shinde and Thomas 2011). The prodomains are most often cleaved, usually by self-processing, after proper folding of the catalytic regions. The pro-regions are generally less conserved than the catalytic part and often contain a very high proportion of basic amino acids, a feature frequently encountered in both inter- and intramolecular chaperones (Winther and Sorensen 1991). Sequence comparisons between SBcas3.3 and the well-studied KP-43 preproprotease (Nonaka et al. 2004) suggested that the observed cleavage might indeed result from autocatalytic maturation of a proprotein composed of the conserved catalytic domain (~510 aa) and a relatively large, non-conserved, prodomain (~260 aa) probably assisting proper folding of SBcas3.3 (Figure 2). The prodomain shows the typical abundance of basic amino acids (theoretical pI for the first 260 aa: ~10.9; for the last 510 aa: ~5.8).

To see if the prodomain is indispensable for protease activity, we produced two N-truncated proteins (cas516 and cas560), one lacking the first 254 amino acids (~ the entire putative prodomain, cas516) and the other, the first 210 (arbitrary chosen, cas560) (Figure 3a), and compared their activities (whole and lysed cells) at several temperatures with that of the whole protease (Additional file 1). Only SBcas3.3 was able to hydrolyse the AZCL-casein substrate, but as already stated, no activity was observed when the producing cells were grown at ≥ 37°C, or when extracts of cells induced at lower temperature (16, 25, or 30°C) were incubated at ≥ 37°C. SBcas3.3 thus seemed to be a cold-active protease that must be produced with its prodomain to be active and forming inactive inclusion bodies when overproduced at 37°C. We suspected, however, that the apparent low optimal temperature might be due to protein instability at low Ca^2+^ concentration, since calcium promotes thermal stability of many subtilisin-like proteins (Siezen and Leunissen 1997). To test this possibility, total extracts were prepared from *E. coli* cells induced at 25°C and their proteolytic activity was tested in the presence of 5 mM additional CaCl_2_. Additional file 1 shows that the optimal temperature of the mature protease shifted upward, but only when the enzyme was pre-incubated with calcium before transfer to higher temperatures, indicating a very low thermal stability in the absence of a sufficient amount of calcium. This ion might also be required for stability of the unprocessed immature protein, which would explain the formation of inactive inclusion bodies at too-high temperature (37°C). The calcium concentration in the cytosol of *E. coli* (< 1 μM) (Jones et al. 1999) is most probably too low to ensure stability of the immature protein, which denatures rapidly and irreversibly at 37°C, while at lower temperatures, its stability is sufficient to allow proper maturation of the enzyme.

### Protein purification

The mature form of the protease was thus purified by Ni-NTA affinity chromatography with calcium (5 mM) added at all steps. This form of the protein was also found exclusively in the insoluble fraction, whatever the growth conditions tested (various low temperatures and IPTG concentrations, osmotic or heat shock (Blackwell and Horgan 1991; Chen et al. 2002)) As sequence analyses did not suggest that SBcas3.3 is a membrane protein, the protease most probably forms aggregates. We thus tried to solubilize these aggregates with the help of urea and detergents, not too concentrated so as not to denature the protein irreversibly. We tested several buffers containing 0.5 M NaCl and 20 ml/l Triton X-100, with urea at various concentrations (0, 0.5, 1, 1.5, and 2 M). A 2 M NaCl solution was also tested. All solutions tested allowed at least partial solubilization of the protein, but best results were obtained with urea at 1.5 M or more. Protease solubilization was thus carried out with 1.5 M urea and 20 ml/l Triton X-100 in the presence of 0.5 M NaCl. SBcas3.3 was next purified according to standard protocols (Figure 3b).

### Biochemical characterization of the purified protease

**Figure 4:**
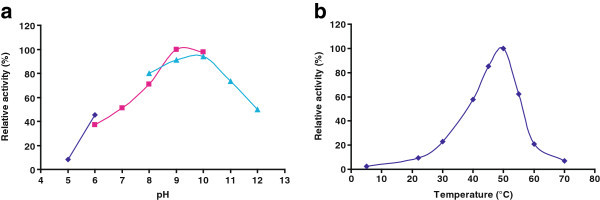
**Estimation of the pH and temperature optima of SBcas3.3. a**. Effect of pH on the relative activity of SBcas3.3. The ability to hydrolyse azocasein was assayed at 50°C in different buffers (♦) 50 mM Na-acetate buffer pH 5–6, (■) 50 mM Tris–HCl buffer pH 6–10, (▲) 50 mM glycine-NaOH buffer pH 8–12. **b**. Temperature-dependent activity of SBcas3.3, measured at pH 9 with azocasein as substrate. No error bars are indicated in these graphs (the standard error of the mean was always below 2%).

The pH and temperature preferences of the purified enzyme were evaluated in the presence of 5 mM CaCl_2,_ with azocasein as substrate. The enzyme proved to be an alkaline protease showing maximal activity between pH 9 and 10. It displayed more than 50% activity between pH 7 and 12 and was active across the entire range of temperatures tested (5-70°C), with an optimum near 50°C (Figure 4).

The calcium dependence of SBcas3.3 was quantified by measuring its relative activity in the presence of CaCl_2_ at various concentrations. For this, the enzyme was pre-incubated at 4°C for 30 min with 0.5 to 20 mM CaCl_2_ before activity measurement. At 50°C, the enzyme activity was maximal at ~4 mM Ca^2+^. As compared to this value, its relative activity was ~33, 50, 71, and 92% at 0.5, 1, 2, and 3 mM CaCl_2_, respectively. To differentiate a direct effect of calcium on protease activity from an indirect one due to possible autoproteolysis during the 30-min pre-incubation at low Ca^2+^ concentration, we transiently removed calcium from the protein by incubation with the chelating agent ethylenediaminetetraacetic acid (EDTA) before estimating its activity in the presence of Ca^2+^ at optimal concentration. Addition of 5 or 10 mM EDTA completely abolished protease activity even at low temperature, and this inhibition was found to be irreversible, as no activity was recovered after CaCl_2_ repletion. This confirmed our preliminary results obtained with cellular extracts: SBcas3.3 is thus strictly dependent on Ca^2+^ for its stability.

To estimate the kinetic parameters of SBcas3.3, the Folin-Ciocalteu method was used to perform initial velocity measurements at various casein concentrations. The maximal velocity (Vmax) and Michaelis affinity constant (Km) were then deduced from a Lineweaver-Burk plot. At its optimal temperature and pH, 1 mg enzyme released 34 μmol tyrosine per min and displayed a Km of 3.8 mg/ml casein.

**Figure 5:**
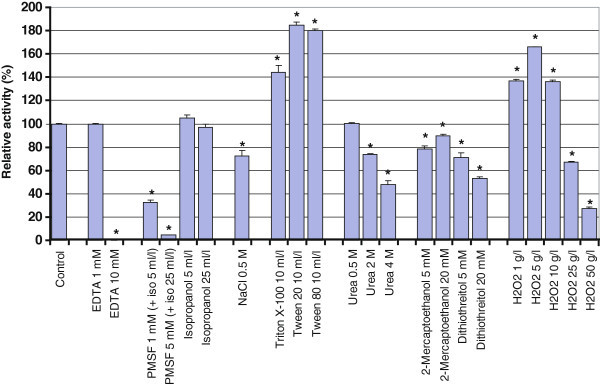
**Effects of various additives on the proteolytic activity of SBcas3.3.** SBcas3.3 was pre-incubated at 30°C for 30 min with each additive before activity measurement at pH 9 and 50°C with azocasein as substrate. *p<0.05 (vs control condition). Error bars represent standard errors of the means of triplicate assays.

The ability of the protease to degrade the azocasein substrate was next assayed in reaction mixtures containing various additives (Figure 5). Proteolytic activity was measured after a 30-min pre-incubation with each of the additives. As expected, SBcas3.3 activity was strongly affected by the serine protease inhibitor phenylmethanesulfonyl fluoride (PMSF). On the other hand, the enzyme was relatively resistant to the denaturing agent urea, still retaining 50% activity in 4 M urea. SBcas3.3 also proved highly resistant to the non-ionic detergents Triton X-100, Tween 20, and Tween 80 and was even activated when one of these detergents was present at 10 ml/l. It was not possible to test the effect of the anionic detergent SDS, which precipitates with Ca^2+^, thus complicating result interpretation. As SBcas3.3 appears related to oxidant-stable proteases, we also tested the effects of reducing agents and oxidants. Interestingly, SBcas3.3 was found to be moderately affected by 5–20 mM dithiothreitol and 2-mercaptoethanol and strongly resistant to high concentrations of hydrogen peroxide. Its activity actually increased when H_2_O_2_ was added at a concentration up to 10 g/l, and 30% activity was still measurable in 50 g/l H_2_O_2_ (1.47 M). SBcas3.3 thus displays characteristics of an oxidant-stable protease.

## Discussion

As compared to other enzymes frequently sought by metagenomic approaches, proteases have rarely been found by functional screening. Most analyses have been done at 37°C with skim milk as substrate to detect proteolytic activity (Lee et al. 2007; Neveu et al. 2011; Pushpam et al. 2011). Here, the agar plates were supplemented with the AZCL-casein substrate from Megazyme and the *E. coli* colonies expressing metagenomic DNA were first incubated at 37°C for 2 days and then for a few weeks at ~22°C. Screening at low temperature is sometimes used to search for cold-active enzymes (Yu et al. 2011), and our study shows it to be useful also for the isolation of meso- or thermophilic enzymes requiring a cofactor for stability. Crystallographic studies have revealed an important structural role for calcium ions in various subtilisin-like proteases, resulting in enhanced thermostability and resistance to autolysis (Briedigkeit and Frömmel 1989; Siezen and Leunissen 1997; Smith et al. 1999). While Ca^2+^ only increases the optimal temperature of many proteases and notably KP-43 (from 60 to 70°C) (Saeki et al. 2002), the biochemically characterized protease to which SBcas3.3 is most similar, its complete removal by treatment with chelating agents irreversibly denatures some enzymes, such as thermitase (Briedigkeit and Frömmel 1989). This is also the case of the newly identified SBcas3.3, which requires CaCl_2_ at ~4 mM concentration for maximal stability.

Another striking difference with KP-43 is that SBcas3.3 is not synthesized with a typical signal peptide and is not secreted into the extracellular medium when overproduced in the heterologous host *E. coli*, even though its identification during the metagenomic screen clearly indicates that at least a small amount of the protein was present after a few days in the growth medium. Several non-secreted proteins have been identified by functional metagenomics, probably as a result of membrane leakage after prolonged growth (Biver and Vandenbol 2013; Phrommao et al. 2011). Its absence in the culture supernatant after one night does not mean, however, that SBcas3.3 is a membrane-anchored or intracellular protease when produced in its original host. It might contain secretion signals not recognized by *E. coli* or by the prediction programs currently available.

Despite these differences between SBcas3.3 and KP-43, easily explainable by the their low similarity (33% identity, 49% similarity), these proteins share several features in addition to the fact that they are produced as large precursors with N-ter parts playing the role of intramolecular chaperones. Both SBcas3.3 and KP-43 have a preference for an alkaline pH (optimum: respectively 9–10 and 11–12) and for relatively high temperatures (optimum: respectively 50 and 60°C). Interestingly, SBcas3.3 is also highly resistant to the oxidizing agent hydrogen peroxide. Most subtilisin-like proteases are rapidly inactivated in the presence of 50 mM H_2_O_2_ (1.7 g/l), notably because of oxidation of a conserved methionine residue adjacent to the catalytic serine (Nonaka et al. 2004). Though SBcas3.3 contains this conserved methionine, it resists H_2_O_2_ concentrations much higher than 50 mM, and its activity is even enhanced up to 10 g/l H_2_O_2_ (0.29 M), in contrast to other oxidant-stable proteases such as AprX from *Virgibacillus* sp. SK37 (Phrommao et al. 2011). The ability to resist oxidizing agents is particularly sought in the detergent industry, which uses oxidants as bleaching agents in an increasing number of products. Several proteases currently used have been genetically engineered to meet this performance requirement, but results are not entirely satisfactory and better enzymes are still sought (Maurer 2004). Though SBcas3.3 meets almost all the requirements for this particular application of proteases (temperature and pH preferences, oxidant stability, detergent resistance), its strict calcium dependence is incompatible with use in most current bleach-based detergents, as these generally contain chelating agents to improve the efficiency of anionic detergents. Crystallographic analysis of SBcas3.3 will help understand the determinants of its marked resistance to detergents, denaturing agents, and oxidants, and should be a useful tool for the rational design of a new protein with a minimized calcium requirement.

## Conclusions

Functional screening of a forest-soil metagenomic library allowed the identification of a novel alkaline serine protease showing only low sequence identity to known proteases. The new enzyme is active over a relatively large pH range and displays high resistance to oxidizing agents, making it particularly attractive for various biotechnological applications.

## Materials and methods

### Construction and screening of forest-soil metagenomic libraries

Two forest-soil metagenomic libraries differing in average insert size (7 and 12 kb) were constructed (Biver and Vandenbol 2013) with metagenomic DNA isolated from a soil sample taken from the A horizon of a Belgian deciduous forest. The sample was collected from the upper 5-cm layer below the litter in Groenendaal at the end of October 2010. Environmental DNA was recovered according to a modified protocol based on the direct extraction method of Zhou et al. (1996), with several modifications (described in Biver and Vandenbol (2013)). Briefly, DNA was extracted by a combination of lysozyme, proteinase K, SDS, and incubation at 65°C. The crude lysate was next purified by chloroform–isoamyl alcohol extraction followed by precipitation with PEG/NaCl. Metagenomic DNA was partially digested with *Sau*3AI, and fragments ranging in size from 4 to 9 kb (small-insert library) and 9 to 20 kb (medium-insert library), recovered after separation in a 1% low-melting-point agarose gel, were inserted into the *Bam*HI-linearized, dephosphorylated pHT01 vector (MoBiTec). The ligation products were introduced into electrocompetent DH10B *Escherichia coli* cells (Invitrogen). About 35,000 *E. coli* colonies from each library (~665 Mb in total) were screened for protease activity by spreading the transformants on 2×YT (2× yeast extract and tryptone) medium (MP Biomedicals) supplemented with 15 g/l agar (Oxoid), 50 μg/ml ampicillin, and 1 g/l AZCL-casein (Megazyme). This substrate releases a soluble blue dye upon hydrolysis. Colonies producing this activity were identified by a blue halo surrounding them. All plates were incubated at 37°C for 2 days and then transferred to room temperature (~22°C) for three weeks.

### DNA sequencing and sequence analysis

The insert encoding SBcas3.3 was sequenced at GATC Biotech (Germany) using the Sanger method. Sequence similarity searches were carried out with the NCBI BlastP program (standard protein blast) and by comparison with proteases of the MEROPS database (http://merops.sanger.ac.uk) (Rawlings et al. 2012). Sequence alignments were performed with the ClustalW program (Thompson et al. 1994) and phylogenetic analysis was done with MEGA5 (Tamura et al. 2011). Signal peptides were predicted with the SignalP 3.0 server (Bendtsen et al. 2004). Theoretical isoelectric points and molecular weights were computed with the ProtParam tool of ExPASy.

### Subcloning in the pET30b expression vector and overexpression in *E. coli* Rosetta 2 cells

The entire coding sequence of the *SBcas3.3* gene was amplified with Platinum *Pfx* DNA Polymerase, 0.5x PCRx Enhancer Solution (Invitrogen), 5’-gggaattc*CATATG*GCGCGCGTATTAAAGGTT-3’ as forward primer, and 5’-cgc*GGATCC*TCATCA**GTGGTGGTGGTGGTGGTG**CGTTCGGCCGACGATCAC-3’ as reverse primer. The restriction sites (*Nde*I, *Bam*HI) used for subcloning are italicized and the histidine tag sequence appears in bold. PCR products were cloned into the pET-30b(+) vector (Novagen) and the resulting plasmids introduced into *E. coli* Rosetta 2 (DE3) (Novagen) after checking the inserts by DNA sequencing. The proteolytic activity of transformants was evaluated by spotting 5 μl overnight culture (grown at 37°C in liquid 2×YT medium, 30 μg/ml chloramphenicol and kanamycin) onto solid 2×YT medium (15 g/l agar) supplemented with 10 μM isopropyl β-D-1-thiogalactopyranoside (IPTG) (Fermentas), 1g/l AZCL-casein, and appropriate concentrations of antibiotics (30 μg/ml chloramphenicol and kanamycin). The bacteria were allowed to grow at 37°C for 16 h before transfer to incubators set at various temperatures (4, 22, 29, 37, and 50°C).

Two N-terminally truncated mutants of SBcas3.3 were obtained similarly, using the same reverse primer as for amplification of the full-length protease and 5’-gggaattc*CATATG*GGATTCAAGATCCTCGATCGTC-3’ (cas560) or 5’-gggaattc*CATATG*GCCATCAAGCGTCCAAGCA-3’ (cas516) as forward primer.

For protein size determination by SDS-PAGE, bacteria were grown for 16 h at 25 or 37°C in liquid Terrific Broth (TB) medium (Difco) containing 10 μM IPTG. Cells of 25-ml cultures were harvested and resuspended in 5 ml lysis buffer (20 mM Tris–HCl pH 8, 5 mg/ml lysozyme). After lysis at 22°C for 30 min and subsequent sonication on ice (4 × 5 s, 100 W), the total extracts obtained were centrifuged (10,000 g, 20 min, 4°C) to separate soluble from insoluble proteins (the latter were next resuspended in 5 ml lysis buffer). Each fraction was mixed with an equal volume of Laemmli’s sample buffer 2× containing 100 ml/l 2-mercaptoethanol and heated at 95°C for 5 min before electrophoresis. Western blot analysis with anti-His tag antibodies was performed as previously described (Matteotti et al. 2012).

### Estimation of the caseinolytic activity of SBcas3.3, cas516 and cas560 on total cell extracts

To better evaluate the activity of SBcas3.3, cas516, and cas560, their ability to degrade the AZCL-casein substrate was also investigated on total extracts of *E. coli* Rosetta cells. For this, bacteria were grown in liquid TB medium with 10 μM IPTG at various temperatures (16, 25, 30 and 37°C) for 16 h (OD_600nm_ ~ 1.5 to 3, depending on the incubation temperature). Total cell extracts were then prepared as described above and diluted twice in 0.1 M buffer (sodium acetate pH 4 to 6, sodium phosphate pH 6 to 8, or Tris–HCl pH 7 to 10) supplemented with 2 g/l AZCL-casein. Incubation was performed on a wheel (2 rpm) at 4, 22, 29, 37, and 50°C until the appearance of the blue colour.

Preliminary information about the calcium requirements of the enzymes was obtained by diluting total cell extracts twice in 0.1 M Tris–HCl pH 9 supplemented with 0 or 10 mM CaCl_2_. This was followed by pre-incubation at 4°C for 0 or 30 min, addition of 1 g/l AZCL-casein, and transfer to 4, 22, 29, 37, or 50°C.

A negative control corresponding to extracts of *E. coli* Rosetta cells transformed with the empty vector was included in all experiments. No activity was observed under any of the conditions tested.

### Purification of the recombinant full-length protease

To purify the recombinant protease, an overnight culture of transformed *E. coli* Rosetta cells was diluted to OD_600nm_=0.005 in a 250-ml shake flask containing 50 ml TB medium and antibiotics (30 μg/ml chloramphenicol and kanamycin). The culture was grown at 37°C under shaking at 160 rpm until the OD_600nm_ reached about 0.4. Overproduction was induced by addition of 10 μM IPTG followed by incubation at 25°C for 16 h. Cells were harvested by centrifugation and lysed at 22°C for 30 min in 20 mM Tris–HCl buffer (pH 8) containing 5 mM CaCl_2_, 0.3 M NaCl, 1 ml/l Triton X-100, and 5 mg/ml lysozyme. After sonication on ice (4 × 5 s, 100 W), the lysate was centrifuged at 10,000 g for 20 min (4°C) to separate insoluble from soluble proteins. The soluble fraction, which did not contain SBcas3.3, was discarded and the insoluble fraction containing the active recombinant protease was resuspended in 20 mM Tris–HCl pH 8, 5 mM CaCl_2_, 0.5 M NaCl, 20 ml/l Triton X-100, 1.5 M urea to solubilize the protease. The lysate was then centrifuged again to collect the soluble fraction now containing the protease, which was next purified by Ni-NTA affinity chromatography (Qiagen). For this, the soluble fraction was first diluted to reduce the urea and Triton X-100 concentrations to 7.5 ml/l and 0.1875 M, respectively, and then incubated overnight at 4°C with 500 μl Ni-NTA agarose pre-equilibrated with binding buffer (20 mM Tris, 5 mM CaCl_2_, 0.5 M NaCl, 7.5 ml/l Triton X-100, 0.1875 M urea, pH 7.5). The resin was recovered by centrifugation at 800 g for 5 min and washed once with binding buffer (flow-through 1) and twice with washing buffer (20 mM Tris, 5 mM CaCl_2_, 0.5 M NaCl, 20 mM imidazole, pH 7.5) (flow-through 2) before elution with 20 mM Tris, 5 mM CaCl_2_,0.5 M NaCl, 175 mM imidazole, pH 7.5 (10 ml). The purified proteins were dialysed at 4°C against 20 mM Tris–HCl pH 7.5 and 5 mM CaCl_2_ and analysed by SDS-PAGE.

### Characterization of the purified protease

Protease activity was estimated by measuring the release of acid-soluble material from azocasein (Sigma-Aldrich). The optimal temperature was determined by incubating 10 μl purified enzyme with 290 μl reaction mix (50 mM Tris–HCl pH 9, 5 mM CaCl_2_, 5 g/l azocasein) at various temperatures from 5 to 70°C. After pre-incubation of the reaction mix, the reaction was started by adding enzyme and stopped after 20 min with 300 μl of 100 g/l ice-cold trichloroacetic acid (TCA). The precipitated proteins were removed by centrifugation at 10,000 g for 10 min (4°C). To increase the sensitivity of the measurement, the supernatant (400 μl) was alkalinized by addition of an equal volume of 2 M NaOH. The mix was centrifuged at 10,000 g for 5 min (22°C) to remove any precipitate before estimation of the azo dye concentration at 440 nm. To determine the optimal pH of the enzyme, protease activities were measured at 50°C over a pH range of 5 to 12 in a mix containing 5 mM CaCl_2_, 5 g/l azocasein, and 50 mM buffer (sodium acetate pH 5–6, Tris–HCl pH 6–10 or glycine-NaOH pH 8–12). The effects of several additives (EDTA, PMSF, isopropanol, NaCl, urea, 2-mercaptoethanol, dithiothreitol, and H_2_O_2_) on protease activity were evaluated by pre-incubating the enzyme with each additive (in 50 mM Tris–HCl pH 9 and 5 mM CaCl_2_) at 30°C for 30 min before incubation with azocasein (in 50 mM Tris–HCl pH 9 and 5 mM CaCl_2_) at 50°C for 20 min. As PMSF has a short half-life at alkaline pH, pre-incubation was done at pH 7.5 instead of 9. The effect of isopropanol was tested because this was the solvent used to prepare the stock solution of PMSF (200 mM) added to the mix. To evaluate the calcium requirements of the purified enzyme, SBcas3.3 was pre-incubated on ice for 30 min with various CaCl_2_ concentrations (0.5 to 20 mM) before measuring its azocaseinolytic activity at 50°C. To see if calcium is required for SBcas3.3 activity or for its stability, Ca^2+^ was removed from the enzyme by pre-incubation with 5 or 10 mM EDTA for 30 min at 4°C before calcium repletion (at 4°C) by dilution in 5 mM CaCl_2_ (final EDTA concentration: 0.67 or 1.33 mM). The residual azocaseinolytic activity of SBcas3.3 was measured after 4 hours of calcium repletion and compared with that of the enzyme pre-incubated without EDTA.

To determine the kinetic parameters of SBcas3.3, 10 μl enzyme was incubated for 20 min at 50°C in 50 mM Tris–HCl pH 9, 5 mM CaCl_2_ and casein at various concentrations (0.1-1 g/l). TCA (500 μl, 110 mM) was then added to stop the reactions (600 μl). The proteins were removed by centrifugation and the amino acids present in the supernatants quantified by the Folin-Ciocalteu method. For this, the supernatants (500 μl) were incubated at 37°C for 30 min with 1250 μl Na_2_CO_3_ 0.5 M and 250 μl Folin-Ciocalteu reagent diluted 10× (Sigma-Aldrich) before absorbance measurement at 660 nm. Activities were determined by comparison with a standard curve of L-tyrosine (0–0.2 mM) and expressed in μmol tyrosine released per min per mg protein. A Lineweaver-Burk plot was used to determine Km and Vmax.

All experiments were performed at least twice in triplicate, and the results of one representative experiment are shown. They are expressed as means ± standard error of the mean. Comparisons with the control condition were performed with Student’s t-test.

### Nucleotide sequence accession numbers

The nucleotide sequence of the insert containing the gene encoding the proteolytic activity was deposited in the GenBank database under accession number KC433657.

## Electronic supplementary material

Additional file 1: **Caseinolytic activity of*E. coli*Rosetta cells overproducing the full-length protease SBcas3.3 or one of its truncated mutants, cas516 or cas560.** (PDF 30 KB)

## Abbreviations

EDTA: Ethylenediaminetetraacetic acid
IPTG: Isopropyl β-D-1-thiogalactopyranoside
Km: Michaelis affinity constant
Ni-NTA: Nickel-nitrilotriacetic acid
OD600nm: Optical density measured at 600 nm
PMSF: Phenylmethanesulfonyl fluoride
SDS-PAGE: Sodium dodecyl sulfate - polyacrylamide gel electrophoresis
TB: Terrific Broth
TCA: Trichloroacetic acid
Vmax: Maximal velocity
2xYT: 2x Yeast extract and tryptone
