# Supplementary material for: Characterization of a new oxidant-stable serine protease isolated by functional metagenomics
Source: Springerplus. 2013 Aug 28;2:410. doi: 10.1186/2193-1801-2-410 (PMC3765597; doi:10.1186/2193-1801-2-410)
Supplement: Supplementary file 1 — Additional file 1: Caseinolytic activity ofE. coliRosetta cells overproducing the full-length protease SBcas3.3 or one of its truncated mutants, cas516 or cas560. (PDF 30 KB) [file 40064_2013_479_MOESM1_ESM.pdf]

Additional file 1. Caseinolytic activity of *E. coli* Rosetta cells overproducing the full-length protease SBcas3.3 or one of its truncated mutants, cas516 or cas560.

Activity was measured with 1 g/l AZCL-casein substrate from Megazyme added to agar plates (whole cells) or to total cell extracts (grown at various temperatures) diluted twice in 0.1 M Tris-HCl pH 9. For the tests on agar plates, the bacteria were first allowed to grow at 37°C for 16 h before transfer to incubators set at various temperatures (4, 22, 29, 37, and 50°C). 10 µM IPTG was used for induction.

							No pre-incubation with $CaCl_2$					Pre-incubation (30 min) at 4°C with 5 mM $CaCl_2$ before activity measurement				
		Caseinolytic activity measured without additional $CaCl_2$					Caseinolytic activity measured with additional $CaCl_2$ (5 mM)					Caseinolytic activity measured with additional $CaCl_2$ (5 mM)				
		4°C	22°C	29°C	37°C	50°C	4°C	22°C	29°C	37°C	50°C	4°C	22°C	29°C	37°C	50°C
**Whole cells (activity evaluated on agar plates)**	SBcas3.3	+/-	+	+	-	-	**not determined**					**not determined**				
	cas516	-	-	-	-	-										
	cas560	-	-	-	-	-										
**Total extracts of cells grown at 16, 25 or 30°C**	SBcas3.3	+/-	+	+	-	-	+	++	+	-	-	+	++	+++	+++	++++
	cas516	-	-	-	-	-	-	-	-	-	-	-	-	-	-	-
	cas560	-	-	-	-	-	-	-	-	-	-	-	-	-	-	-
**Total extracts of cells grown at 37°C**	SBcas3.3	-	-	-	-	-	-	-	-	-	-	-	-	-	-	-
	cas516	-	-	-	-	-	-	-	-	-	-	-	-	-	-	-
	cas560	-	-	-	-	-	-	-	-	-	-	-	-	-	-	-
